# Direct *in situ* observation of metallic glass deformation by real-time nano-scale indentation

**DOI:** 10.1038/srep09122

**Published:** 2015-03-16

**Authors:** Lin Gu, Limei Xu, Qingsheng Zhang, Deng Pan, Na Chen, Dmitri V. Louzguine-Luzgin, Ke-Fu Yao, Weihua Wang, Yuichi Ikuhara

**Affiliations:** 1 WPI-Advanced Institute for Materials Research, Tohoku University, Sendai 980-8577, Japan; 2 Institute of Physics, Chinese Academy of Sciences, Beijing 100190, China; 3 International Center for Quantum Materials, Peking University, Beijing 100084, China; 4 Collaborative Innovation Center of Quantum Matter, Beijing 100871, China; 5 School of Material Science and Engineering, University of Shanghai for Science and Technology, Shanghai 200093, China; 6 School of Materials Science and Engineering, Tsinghua University, Beijing100084, China; 7 Institute of Engineering Innovation, School of Engineering, The University of Tokyo, Tokyo 113-8656, Japan

## Abstract

A common understanding of plastic deformation of metallic glasses (MGs) at room temperature is that such deformation occurs via the formation of runaway shear bands that usually lead to catastrophic failure of MGs. Here we demonstrate that inhomogeneous plastic flow at nanoscale can evolve in a well-controlled manner without further developing of shear bands. It is suggested that the sample undergoes an elasto-plastic transition in terms of quasi steady-state localized shearing. During this transition, embryonic shear localization (ESL) propagates with a very slow velocity of order of ~1 nm/s without the formation of a hot matured shear band. This finding further advances our understanding of the microscopic deformation process associated with the elasto-plastic transition and may shed light on the theoretical development of shear deformation in MGs.

Since its discovery^1^, bulk metallic glasses (BMGs) have distinguished themselves from their crystalline counterparts^2^,^3^,^4^ due to their ultrahigh strength and hardness with promising applications in various fields. Nevertheless, the Achilles' heel of BMGs, e.g., the low tensile ductility^5^, greatly hinders their engineering performance in general. Because shear bands provide preferential sites for the ongoing plastic flow responsible for most catastrophic failures, it is widely thought that the highly localized shearing and resultant strain softening are the main causes for the low ductility of BMGs^6^,^7^,^8^. To tackle this issue, tremendous efforts have been devoted to developing BMGs composites by introducing ductile crystalline phases^9^. In addition to the BMG composites, small MG samples subjected to tensile tests also show promising plasticity in a manner via both homogeneous and inhomogeneous flow without catastrophic failure^10^. This has stimulated intensive interest in disclosing the basic physics behind the microscopic deformation mechanisms of MGs from both theoretical and experimental aspects^11^,^12^,^13^,^14^,^15^,^16^,^17^,^18^,^19^,^20^. In principle, the ductility of MGs, regardless tensile or compression, is ascribed to the activation of more shear transformation zones (STZs) and more homogeneous distribution of STZs. However, an experimentally determined microscopic picture associated with this deformation process remains lacking.

Nowadays in-situ nanoscale mechanical experiments of metallic materials in a transmission electron microscope (TEM) can dramatically improve the spatial resolution of the deformation process that may help to advance the understanding of the intrinsic characteristics of BMG deformation^21^. Using this technique, here we demonstrate that multi-step type plastic events do occur within an elastic deformation regime of a MG. The most distinct characteristic of such deformation is the lack of matured shear bands, which is facilitated by stable shear events. We present our results from both in situ nanoindentation of a Zr-based MG within a transmission electron microscope (TEM) and theoretical simulations to witness the unusual phenomenon.

Since the TEM-NanoIndenter system (Nanofactory, Goteborg, Sweden) provides a unique opportunity to picture the nanoindentation process in real time within a TEM and simultaneously to acquire the force-displacement data, we applied the technique to deform MGs with a thickness less than 50 nm inside a JEOL 2010F TEM (JEOL, Tokyo, Japan) as shown in Fig. 1(a). Snapshots of the entire deformation process were recorded by a TV rate camera (25 frames per second) to calculate the force-displacement curve.

As can be seen from one of the typical force-displacement curves measured *in situ* on a 50 nm-thick Zr_62.5_Cu_22.5_Fe_5_Al_10_ MG shown in Fig. 1(b), two separate deformation events were detected within the loading process. The one with an identical slope as in the unload curve's can be attributed to a pure elastic deformation, while the ones with a horizontal part represent a “pop-in”-like plastic deformation when major strain softening occurs. Compared to the reported “pop-in” events that completed instantaneously associated with the initiation of shear bands^11^,^22^, the plastic event here arises in a rather sluggish way, namely 6 s for a 30 nm displacement, as revealed by the *in situ* image recording during the indentation.

Figure 2 shows 6 s consecutive snapshots corresponding to the above “pop-in” process in the thin foil at a deformation rate of 5 nm/s. Such a deformation speed is much lower than the typical shear band velocity in bulk (massive) glassy alloys^23^. Finite element modeling analysis in Fig. 2(a) reveals that most stress concentrates mainly close to the indenter tip region. We note that only half of Fig. 2(a) and Fig. 2(b) is shown due to mirror symmetry with the dashed line marking the tip position. The sudden emergence of the darker region close to the indenter corresponds to the initiation of the “pop-in” phenomenon, which displays a real-time description of the microscopic deformation process. As can be seen from Fig. 2(c)–2(h), the deformed region gradually evolves into a new-moon-shaped zone around the tip area, in contrast to that at the initial stage shown in Fig. 2(b). After retraction, the moon-shaped contrast remains [Fig. 2(i)], indicating that the “pop-in” like phenomenon indeed corresponds to irreversible plastic deformation via visible structural morphology transition. The diffraction pattern of the deformed region [inset of Fig 2(i)] confirms the amorphous morphology. It is important to stress here that the geometry of the dark region is nicely consistent with the stress-confined area depicted in Fig. 2(a), implying that the plastic deformation occurs mainly in the vicinity of the indenter tip without any sign of shear band formation.

We also investigate the deformed region of the same specimen using SEM method after the nanoindentation tests (see Fig. 3a). The deformed region becomes much thicker than the original thickness of ~50 nm (Fig. 3b), indicative of a significant expansion in the out-of-plane direction. More interestingly, there are a few liquid-like beads in the contact region indicated by the yellow arrows (Fig. 3b), which are typically observed on the fracture surface of BMGs accompanying a temperature rise due to transient energy dissipation^24^. This indicates that the viscosity of the deformed region beneath the indenter may become significantly low^25^.

In situ nanoindentation within a TEM applied in our experiments offers the opportunity to spatiotemporally resolve the evolution of plastic flow in the Zr-based MGs that is difficult to access using conventional macroscopic indentation tests. The force-displacement curve of the Zr-based MG clearly illustrates an elasto-plastic response with a few discrete plastic events leading to irreversible deformation at ambient temperature. Moreover, the plastic flow evolves in a much slower speed of the order of ~1 nm/s in contrast to runaway shear bands propagation^26^,^27^. Although shear-banding dynamics analysis indicates that shear band can remain cold and slide in a stick-slip manner^28^, the propagation velocity in the present study is still much lower than the reported values (~1 μm/s) for those cold shear bands. It is suggested that this flow zone may be an “embryo” of a matured shear band and is thus more stable^27^. Regarding the microscopic deformation mechanism of MGs, it implies that the fundamental unit process underlying deformation must be a local rearrangement of atoms involved in shear transformation zones (STZs) that can accommodate shear strain^29^. Since no shear band is observed in the present study, the plastic deformation is suggested to be facilitated by STZs which are activated and highly connected together to form the relatively stabilized embryonic shear bands (ESBs).

Usually, such STZ-mediated plastic deformation behavior can be observed at either elevated temperatures near *T_g_* or at high strain rates^6^. However, neither condition can be applied to our case. Firstly, during the nanoindentation tests, the estimated temperature rise is as low as 1 K^30^. Secondly, According to *dε*/*dt* = 1/*h* × *dh*/*dt*, the strain rate is calculated as ~2 × 10^−3^/s, much lower than the value that can induce homogeneous plastic flow. Previous computational simulations and experimental study on nanoindentation tests reported inhomogeneous deformation in terms of shear banding beneath the nanoindentation tip^6^,^31^. However, the further evolution of shear banding is suppressed in the present study. Several indentation studies on the deformation mechanisms of MGs also mentioned that shear bands, in some special cases, cannot be detected around the indent on the specimen surface^32^,^33^,^34^,^35^,^36^,^37^. For a tough Zr_52.5_Al_10_Ni_10_Cu_15_Be_12.5_ MG^32^, a further investigation of the deformation regions underneath the indent reveals the formation of dense shear bands despite the absence of shear bands near the indent on the surface. This implies that the Zr-based MGs are capable of generating multiple shear bands but exert a high resistance to the propagation of these shear bands to the surface due to the constrained condition in the indention tests. Besides, both the surface irradiation and the annealing treatment can induce structural modification, especially on the surface stress state and the amount of the excess free volume^33^,^34^,^35^,^36^,^37^. Reduction of free volume is supposed to increase the resistivity to the nucleation and propagation of shear bands, thus no shear band forms around the indenter in the annealed metallic glasses^34^,^35^,^36^,^37^. In addition, the overall deformation seems homogeneous underneath the indenter. The present experimental setup is advantageous over the macroscopic indentations since the microscopic deformation process can be clearly captured. Although the experimental condition in this study is different from the macroscopic indentation, the basic physics behind this phenomenon is similar. In both cases, plastic deformation proceeds in terms of many localized shear events whereas the percolation of these shear events is suppressed, thus a delay in nucleation and rapid propagation of one main shear band occurs. We attribute this to the geometrical factor of our sample with thickness of ~50 nm. Shimizu et al. reported an aged-rejuvenation-glue-liquid (ARGL) model of shear band in BMGs in which the stressed material region is required to surpass an incubation length scale *L_glue_* for developing a matured shear band from STZs^38^. *L_glue_* is defined as:
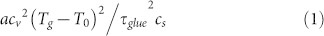
where *α* is the thermal diffusivity of the MG, *c_v_* is the volumetric specific heat, *T_0_* is the ambient temperature, *τ_glue_* reflects the rate of recovery, and *c_s_* is the shear wave speed. *L_glue_* of Zr-based BMGs is then predicted as ~100 nm^38^. As shown in Fig. 3c, due to the smaller size involved in the deformation than *L_glue_*, the deformed sample only has the glue zone (zone 1), rejuvenation (zone 2) and the elastic region (zone 3). In this case, the specimen should have many ESBs but no major shear band. The deformation proceeds in a quasi steady-state localized shearing manner. In addition, there is no geometrical confinement along z-axis. The out-of-plane degree of freedom provides a diffusive channel through which the stressed atoms can move leading to relaxing of the “jammed” high energy state^39^. This agrees well with both the experimental and simulation results. Experimentally, the ultimate deformation region shown in Fig. 2i becomes darker but is not extended any more compared to that (Fig. 2h) formed after the first “pop-in” like plastic deformation. It confirms that during the deformation, the atoms with increased mobility mainly move upward along the z-axis.

To assist the understanding of the mechanical process for plastic deformation, molecular dynamics (MD) simulation was performed using LAMMPS package^40^. To simplify the calculation, our system consists of 4000 atoms at a composition of Zr_50_Cu_50_ and 1323 atoms in a fixed substrate with a dimension of 41 Å × 50 Å × 41 Å. The sample has periodic boundary conditions (PBC) in x-axis, free surface in z-axis and a substrate in y-axis. To test the validity of the simulation with slight chemical fluctuation, we also performed simulation with a single elemental component, which shows no difference from that shown in Fig. 4. The initial state was prepared by a fast cooling to 300 K by avoiding crystallization. Spherical indenter with a radius of 8 Å is used to indent at the top center of the sample along y-axis at a constant loading speed of 0.02 Å/ps. We illustrate the initial configuration before loading in Fig. 4(a), in which the sample has a relative flat surface on top of y-surface. After 1 ns shown in Fig. 4(b), a major expansion in z-axis, along the free surface direction, is observed with a pronounced squeezing in y-axis due to indentation. This deformation behavior shows dramatic similarity with that revealed by our in situ experiments in Fig. 2. The corresponding force-time curve is shown in Fig. 4(c). Diverged deformation behaviors in the z-free and PBC directions are observed due to different boundary conditions. The atomic mobility is lowest in x-axis becaused of the infinite dimensions, consequently the atomic motion in z-axis is more than that in x-axis when y-axis is supressed since the z-axis consists of free surfaces. The insets are the side-views of the deformed samples. The stretched or compressed atoms can easily release the stress upon driven to free surfaces. Therefore, the accumulated elastic energy can be released before the critical fluctuation biasing the energy barrier is sufficient enough for shear band propagation. Accordingly, the plastic deformation proceeds via collective movement of STZs or ESBs without any shear band.

Previously, inhomogeneous deformation was attributed to shear-band-mediated plastic events and plastic deformation via shear events without developing a shear band is barely considered. Furthermore, absence of shear bands is typically regarded as a sign of homogeneous deformation occurring in metallic glasses. For instance, recent reports^17^,^18^,^41^ on elastostatic compression-induced structural disordering show that homogeneous deformation of MGs can be achieved at a stress below its global yield strength due to local entropic fluctuations via cooperative atomic rearrangement. Similar experiments were also performed in a model of BMG, ZrTiCuNiBe, at a load of 80% of the yield stress, indicative of a large expansion in volume associated with shear events in the glass^42^. The above experiments suggest that free volume can be generated and homogeneously distributes in a matrix without any shear band. On the other hand, Greer et. al. demonstrated a strong-yet-brittle to a stronger-and-ductile transition by size reduction of MGs^43^,^44^. They also reported that homogeneous deformation prevails over crack-like shear-band propagation at 100 nm diameter^43^. Note that the fracture of the MG still takes place via shear failure as opposed to drawing-to-a point observed in homogeneously deformed metals, implying that the shear banding mechanism is not completely surpressed^43^, consistent with our results. This indicates that the formed ESBs can be very stable, and thus do not develop into highly localized matured shear bands despite that the macroscopic deformation seems homogeneous.

In conclusion, we report the plastic deformation of MGs indeed occurs without the formation of any shear band. The plastic flow proceeds very slowly at the early embryonic stage of shear band formation at ambient temperature. This work advances our understanding of the microscopic deformation process related to the elasto-plastic transition before nucleation of a matured shear band, which sheds light on the theoretical development of shear deformation mechanisms in MGs.

## Methods

The master ingot of the Zr_62.5_Cu_22.5_Fe_5_Al_10_ alloy was prepared by arc melting mixtures of Zr, Cu, Fe and Al elements with purity of 99.9%, 99.99%, 99.9% and 99.99%, respectively, in a high purity argon atmosphere. Ribbon samples with a cross section of 0.02 × 1.2 mm^2^ were prepared by melt spinning. The glass transition temperature *T_g_* of the ribbon samples is 651 K. In order to achieve a more homogeneous and relaxed glassy state, the glassy ribbons encapsulated in quartz tube under a vacuum were isothermally annealed at 713 K for 300 seconds, and quenched into water subsequently. To exclude the influences from Ga ion implantation, we have taken a conventional TEM sample preparation technique by dimpling instead of focused ion beam, which was widely used nonetheless, followed by an Ar gentle milling at low energy of 2.0 keV with a 0.5 keV finish.

## Author Contributions

L.G. and N.C. designed the project. L.G., Q.S.Z. and N.C. carried out the experiments. L.M.X. conducted the computer simulations. D.P. did the finite-element modeling analysis. L.G., L.M.X., Q.S.Z., D.P., N.C., D.V.L., K.F.Y., W.H.W. and Y.I. analyzed the data. L.G. and N.C. wrote the manuscript.

## Figures and Tables

**Figure 1:**
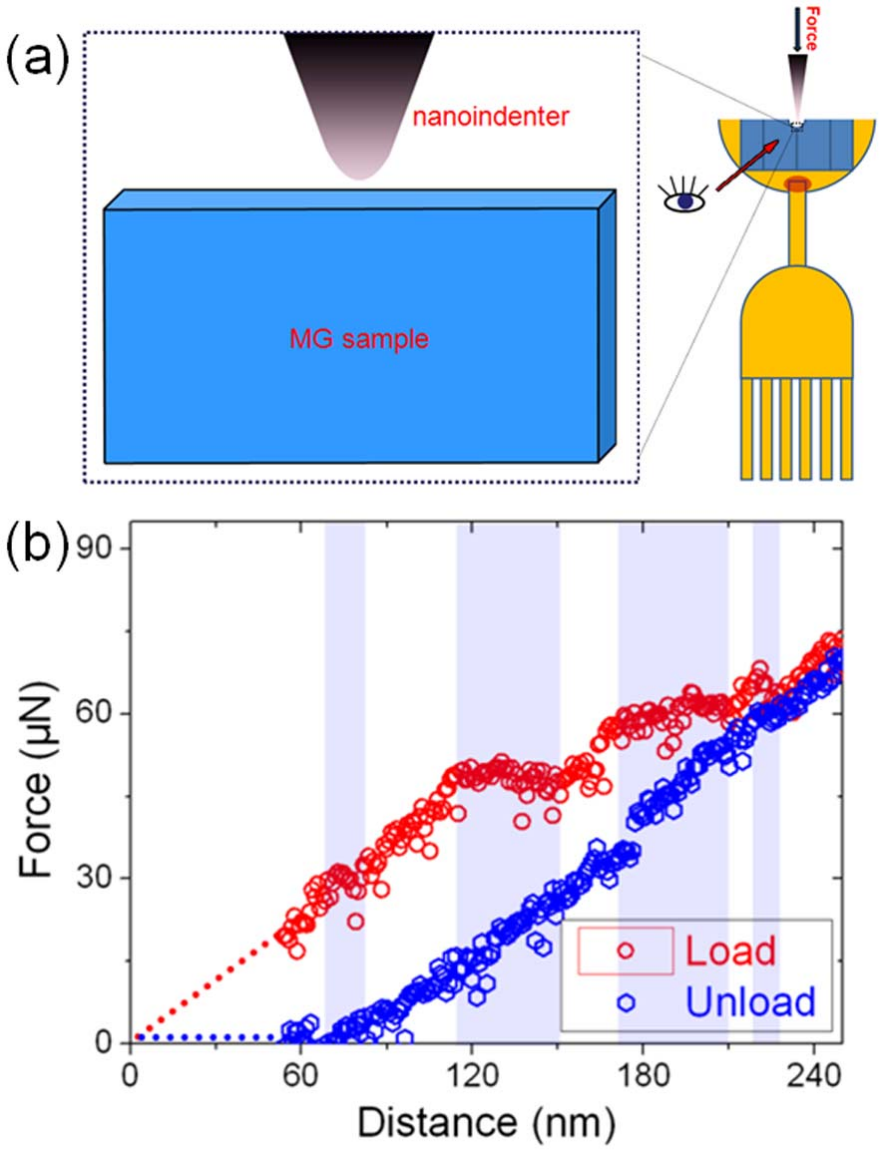
The experimental set-up and the typical data obtained from the nanoindentation tests. (a) The schematic diagram of the nanoindentation test, (b) one of the typical force-displacement curves measured *in situ* on a 50 nm-thick Zr_62.5_Cu_22.5_Fe_5_Al_10_ MG. Four separate plastic events were detected within the loading process, including two small load drops and two displacement bursts as highlighted.

**Figure 2:**
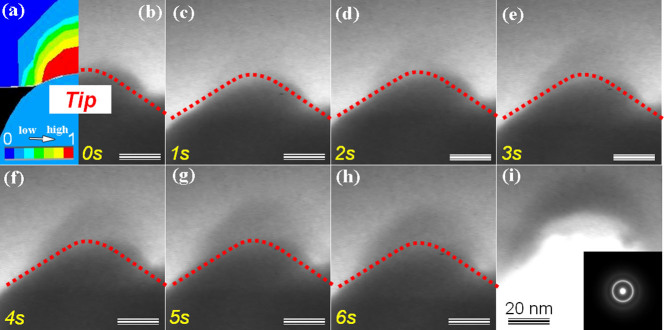
Dynamical snapshots of the deformation process. (a) the image prior to nanoindentation with the finite-element modeling analysis shown in the inset, (b)–(h) Dynamic snapshots of consecutive 6 s within one “pop-in” process, (i) the image after retraction indicates that the new-moon-shaped contrast remains. Note that the dashed lines were extrapolated for better visualization.

**Figure 3:**
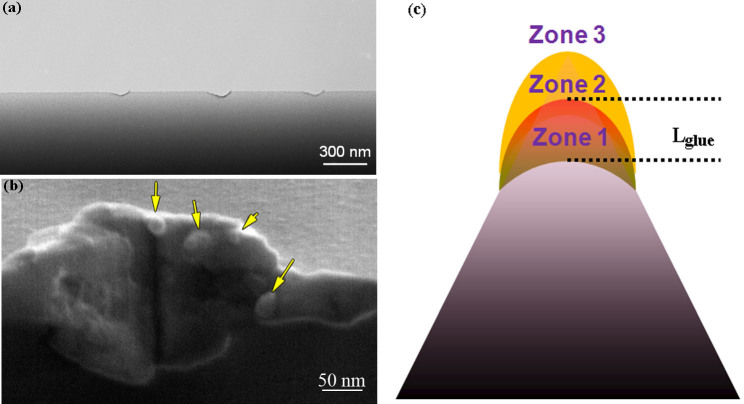
Characterization of the deformed samples after the nanoindentation tests. (a) SEM images of the deformed samples, (b) the enlarged nanoindenter, and (c) Schematic diagram of the deformed region based on the ARGL model. Zone 3 is elastic region, Zone 2 is rejuvenation region and Zone 1 is glue zone.

**Figure 4:**
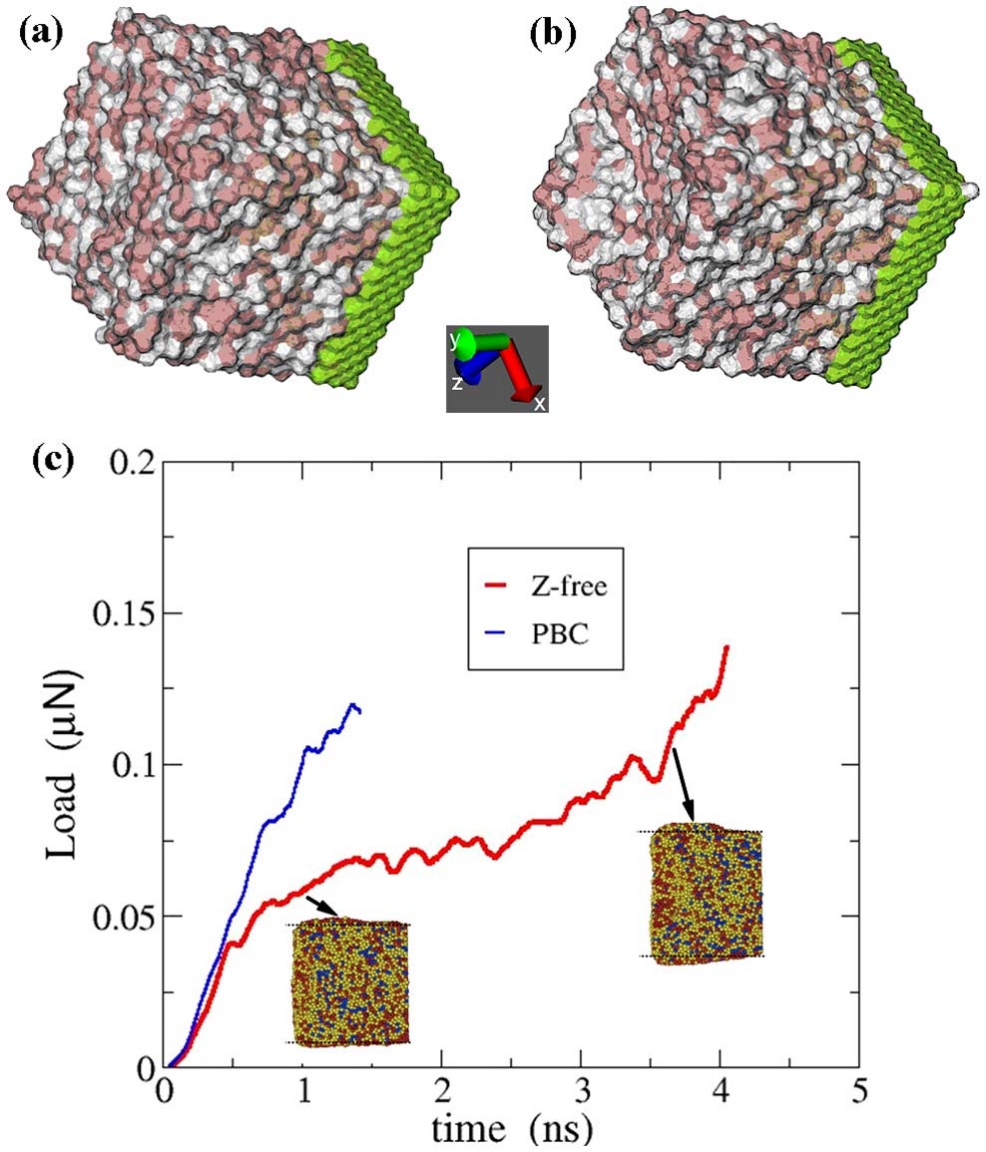
MD simulation of the nanoindentation test. (a) the structural configurations before indentation, (b) the simulated sample deformed after the indentation for 1 ns, and (c) the corresponding force-time curve. The insets are the side views of the deformed sample.
